# Focus stacking: Comparing commercial top-end set-ups with a semi-automatic low budget approach. A possible solution for mass digitization of type specimens

**DOI:** 10.3897/zookeys.464.8615

**Published:** 2014-12-16

**Authors:** Jonathan Brecko, Aurore Mathys, Wouter Dekoninck, Maurice Leponce, Didier VandenSpiegel, Patrick Semal

**Affiliations:** 1 Royal Belgian Institute of Natural Sciences, Vautierstraat 29, B-1000 Brussels, Belgium; 2 Royal Museum for Central Africa, Tervurensesteenweg, Tervuren, Belgium

**Keywords:** Focus Stacking, Cognisys, StackShot, Leica, Zerene Stacker, Helicon Focus, mass digitization

## Abstract

In this manuscript we present a focus stacking system, composed of commercial photographic equipment. The system is inexpensive compared to high-end commercial focus stacking solutions. We tested this system and compared the results with several different software packages (CombineZP, Auto-Montage, Helicon Focus and Zerene Stacker). We tested our final stacked picture with a picture obtained from two high-end focus stacking solutions: a Leica MZ16A with DFC500 and a Leica Z6APO with DFC290. Zerene Stacker and Helicon Focus both provided satisfactory results. However, Zerene Stacker gives the user more possibilities in terms of control of the software, batch processing and retouching. The outcome of the test on high-end solutions demonstrates that our approach performs better in several ways. The resolution of the tested extended focus pictures is much higher than those from the Leica systems. The flash lighting inside the Ikea closet creates an evenly illuminated picture, without struggling with filters, diffusers, etc. The largest benefit is the price of the set-up which is approximately € 3,000, which is 8 and 10 times less than the LeicaZ6APO and LeicaMZ16A set-up respectively. Overall, this enables institutions to purchase multiple solutions or to start digitising the type collection on a large scale even with a small budget.

## Introduction

Since the first photographic equipment was developed, people have tried to record natural history specimens with their equipment. This has always worked for regularly sized objects; however, the micro-world remained unrecorded for a long time. When suitable lenses made it possible to capture small creatures on film it was clear that other problems arose. The low depth of field made it almost impossible to get the complete object in focus, unless the aperture was stepped down (Tindall and Kalms 2012). However this resulted in other aberrations as the optical resolution reduces due to the diffraction effect (Davies 2010, Gallo et al. 2014, Goldsmith 2000). As computers have become widely available, numerous software have been developed by microscope companies and researchers, making it possible to combine pictures with different depths of field to create a focus stack in which the entire object is in focus (Adelson et al. 1984, CombineZP (www.hadleyweb.pwp.blueyonder.co.uk), Helicon Focus (www.heliconsoft.com/heliconsoft-products/helicon-focus), Auto-Montage (www.syncroscopy.com/Auto-Montage), Zerene Stacker (zerenesystems.com/cms/home)). In the beginning this technique was only available for laboratories or research groups with a large budget to spend on a state of the art stacking column or microscope, with special lenses, lighting, stage control, camera and software. Although results were better than a single image, the system itself was sometimes difficult to operate without a training period. These systems could be relatively fast, but often, post-processing was time consuming and most importantly, as techniques change rapidly, an update on the hardware of these systems is quite expensive.

The way to determine if a picture of a specimen is according to the right parameters and can be considered to be a ‘good’ and ‘useful’ picture is when the following settings have been met: i) the image needs to have an in-focus specimen; ii) there shouldn’t be any parts that are over-exposed or under-exposed; iii) all parts that are necessary to identify/study a specimen in a specific view have to be visible and distinguishable. As we live in a time where everything is digitally accessible, these last parameters might only be met when viewing the image at its full size at the highest resolution and not in the printed version within an article or a book. Secondly and equally important to the other previously defined parameters, these pictures need to be taken as fast as possible and the post-processing time needed to get a publishable, usable picture has to be as low as possible. If these different parameters are met, the system can be used to provide pictures for an occasional publication, and also for the mass digitization of type material. This is very important in collection management because in some cases digitization could replace the need to ship or send very fragile specimens for study in all kinds of disciplines.

The high resolution multimedia recording of small specimens is a real challenge for Natural History museums who are working on mass digitization programs. The quality of the resulting image, the cost of the equipment, the human work and the learning curve are important parameters in order to define a general digitization strategy.

We present here a low budget-high quality approach consisting of commercial products. We will compare different software packages using the pictures produced from this set-up. In addition to this comparison we will have a closer look at several available high-end solutions regarding focus stacking and compare them to our set-up.

As it is important to compare the cost of purchasing the different techniques we will give a relative quotation (due to the confidentiality of price quotations) based on past purchase prices and recent received quotations. This will give an indication about the price range of the different stacking solutions.

## Material and methods

### Choice of the tested specimens

We chose two different specimens for the tests in this paper. The first is an ant from the genus *Meranoplus*, whilst the second is a beetle of the genus *Trachys*. Both specimens represent a challenge to obtain a decent picture. The main challenge for the ant specimen is that it has lots of hairs, it is reflective and has fine long body parts. The beetle does not have a lot of details, but the curvature and the brilliance of the elytra makes it very difficult to get an evenly exposed specimen, as they tend to reflect a lot of light. Only the ant will be used to compare the different stacking software, whilst both of the specimens will be used to check the different solutions for creating a focus stack. The reason we did not chose both specimens for each comparison is that for the creation of the stacked image the ant will be the most difficult as small intersecting details like hairs tend to create halos during the stacking process. However the beetle will not create such a problem, therefore, it is not necessary to use this specimen in the visual software comparison. Both specimens are interesting to consider in the solution section because this enables the comparison of lighting, stability of the system, etc.

### Canon-Cognisys set-up

**Figure 1.**
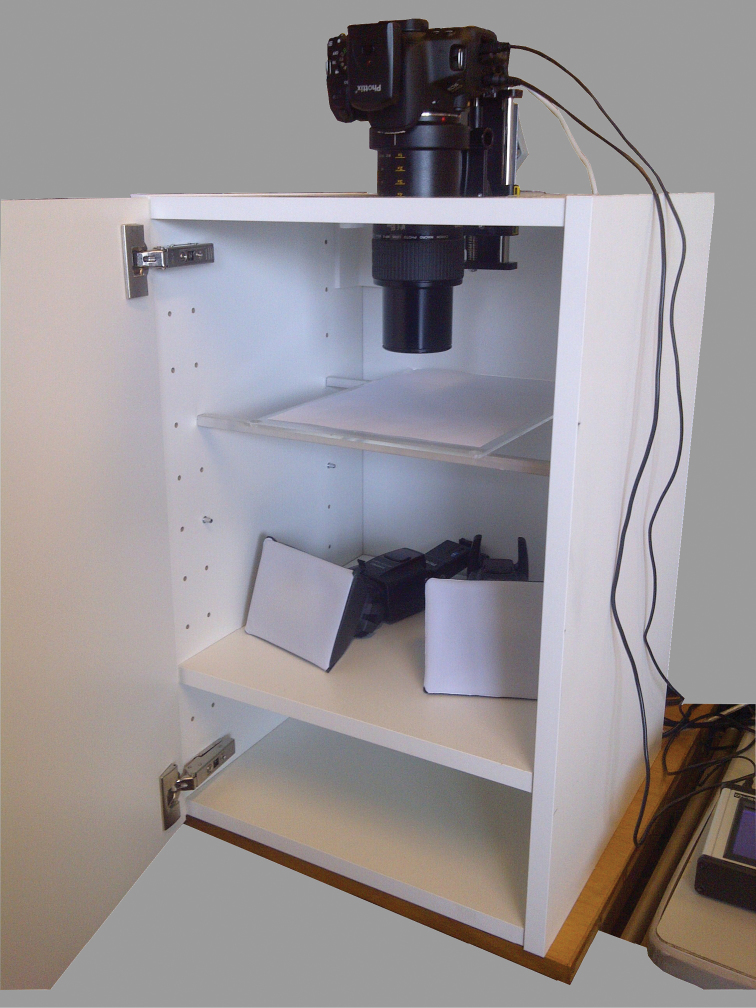
The Canon-Cognisys set-up at the RBINS. The Canon 600D Camera, equipped with a Canon MP-E 65 mm 1:2.8 1–5× Macro Photo Lens, is mounted on the vertically positioned StackShot (Cognisys inc.). In the Ikea closet, at the bottom, you see the two Yongnuo YN560II speedlites positioned frontally and above is the plexiglas fig covered with paper to position the specimen.

We got the main idea for this set-up from the set-up developed by Dr. Anthony G. Gutierrez and Graham Snodgrass, which is described by Alexander and Droege (Sine Dato) from the USGS Bee Inventory and Monitoring Laboratory (BIML 2010). In this set-up we use a Canon EOS 600D camera with a resolution of 18 MP in ‘large’ picture mode. The camera is equipped with a Canon MP-E 65 mm 1:2.8 1–5× Macro Photo Lens (Figure 1). This lens starts where other macro lenses end, at 1:1, and is able to magnify the object 5×. We used two low budget flash lights (Yongnuo Flash YN560-II). One flash is controlled by a remote (Phottix PT-04 II), while the other works in the auto-slave mode to flash in synchrony. Both the specimen and the flash lights are positioned inside an Ikea kitchen closet (Metod, 40 × 60 cm, Häggeby White) with a removable background. This background was neutral grey for this test, but can be any colour desired. The flash lights are positioned away from the specimen. To automate control when taking the different images, we used a Cognisys StackShot which drives the camera from the set beginning to end positions, taking pictures every programmed number of microns. The StackShot is positioned vertically on top of the Ikea closet in which a hole is cut out to fit the camera and StackShot. The StackShot holds the camera and is attached to a metal reinforced corner.

We used the auto distance function (Auto-Dist) of the StackShot controller for the tests and did so for most of the stacking used in this set-up. In this way a certain step size in µm was chosen according to the size of the object and the magnification used. The f-stop is chosen depending on the magnification. Up to a magnification of 2×–3× we use an f-stop of 5.6 or 5.0, while a magnification of 3× to 5× was followed with an f-stop of 5.0 or 4.5 and sometimes 4.0, depending on the specimen photographed. The flash lights were set at a light intensity of 1/64th to 1/4th of the flash power for the smallest specimens. The StackShot controller triggers the camera through a shutter speed cable. Choosing the beginning and the end positions of the stack is done by means of the Live View function in the Canon Eos Utility software package for remote shooting. An additional LED light is positioned in the closet to light the specimen during the setting of the beginning and end positions. The Led light is switched on/off by a USB controlled power plug (USB Net Power 8800 by Aviosys, www.aviosys.com/8800.html). The StackShot itself can be controlled in Zerene Stacker, the stacking software, as well, but we preferred to do this directly on the StackShot.

### Comparison of the software packages

To compare a few of the software packages available we chose one set of pictures produced by the Canon-Cognisys setup described above. We recorded the time to process the stack of pictures and looked in detail at the quality of the stacked image. When addressing the details available in the pictures it is best to download the full resolution images as most of the details discussed were not visible on the downscaled pictures – http://mars.naturalsciences.be/publications/zookeys. All the tests below were performed using the default settings of the software packages.

### Auto-Montage

Auto-Montage is a stacking software program by Syncroscopy. This was one of the first software packages commercially available to perform focus stacking. It offers different ways to stack a set of images (fixed, blended, weighted, exponentially weighted and compound weighted). As well as this choice, the software has the possibility to align a stack of images before the stacking procedure starts. We used an evaluating version for this test. This made it rather difficult to look at fine details as the picture is imprinted with watermarks. However, it was possible to get a time indication and allowed an overall view of the stacked image. The maximum number of pictures that are possible to load into one stack is 255 pictures. However, it is advised to make sub-stacks of such a large focus stack in other programs as well (cf. Zerene Stacker).

### CombineZP

CombineZP is widely used and one of the pioneer software packages to create an extended focus image. Therefore we tested this software package which is freely available. CombineZP has multiple options to stack a set of pictures (Do Stack, Do Soft Stack, Do Weighted Stack, Pyramid Weighted, Pyramid Do Stack, Pyramoid Maximum Contrast). There are also two ways to align a stack of pictures. One is the quick alignment, while the other one is the thorough alignment. CombineZP offers a batch process program also, so if it is more suitable to use slower stacking methods, these can be used as a batch overnight. The only downside is the poor memory allocation of the software so a more powerful workstation is needed for larger and more extensive sets of images.

### Helicon Focus

Helicon Focus is another commercially available stacking software program, produced by Helicon Soft. It has a straightforward interface and enables the user to retouch the pictures after stacking in the Pro version. Helicon Focus offers three possibilities to stack a set of pictures. These are called method A, B and C, which are an average, depth and pyramid stacking method respectively. The maximum number of pictures that are possible to load into one stack is 255 pictures.

### Zerene Stacker

Zerene Stacker (Build T201404082055) is a commercially available image stacking software package, created by Zerene Systems, which enables the user to retouch stacks within the program when necessary. In Zerene Stacker there are two possibilities to stack a set of images, PMax and DMap. The main difference between the two stacking techniques is that PMax handles a large number of images per stack really well. But PMax can alter colour and contrast from what appears in the original unstacked source images. This behavior is common to all the comparable pyramid methods. The shifts are mostly a slight loss of saturation and increase of contrast. In addition, different specimens often have slightly different colors and those colors may have faded in storage. The DMap option will create better-looking pictures but creates halo effects when too many pictures are stacked using this option. If the morphology of the specimen pictured is more important than the colour then it is better to choose the PMax method. This is generally the case for dried specimens, which have lost colour partially over time due to storage. When dealing with fresh material and colour is equally important, then DMap might be a better stacking option; however, calibration of the lighting setup is necessary in every step of the process. Therefore the best option is to combine both techniques and create substacks using PMax and stack the resulting substacks by employing DMap. But these settings are necessary only when it comes to a large (>100) number of pictures. This can all be done by using the automatic sub-stacking program written by Chris Slaybaugh (https://dl.dropbox.com/u/51805918/SlabberJockey--V1.0.zip). In this test we provide the results only for both stacking methods made without sub-stacks. Specimens with many fine structures, such as hairs, benefit from the sub-stacking technique to achieve sharpness without halos.

### Comparison of the high-end focus stacking solutions

We used two high-end approaches that were already available in-house, a Leica MZ16A set-up and a Leica Z6APO. These solutions will be compared to the Canon-Cognisys set-up we describe above.

### Leica MZ 16 A with DFC500

The Leica MZ16A microsystem was equipped with a DFC500 camera. This camera is able to make pictures with a total size of 12 MP (4080 × 3072). The objective used is a 0.63× Leica Planapo. The lights used are two lights controlled by a Leica KL 1500 LCD. The software controling the Z-stage is LAS Core by Leica. The aperture was set to its maximal opening. The exposure time was set according to the object and distance of the lights. Because two direct lights were used, the light needed to be diffused. This was done by using chalk paper and/or opaque plastic (Leica, S.D.).

### Leica Z6APO with DFC290

The Leica Z6APO macroscope tested is equipped with a DFC290 camera, which is able to take pictures of 3 MP. The objective used was the Leica Planapo 2.0×. The lighting used in this set-up were two Manfrotto ring systems consisting of 24 LEDs each. They are opposed to each other and a diffuser is set within the ring. This entire set-up was set over the specimen. The aperture was set to its maximal opening. The exposure time was positioned according to the colour and reflectivity of the specimen. Setting the start and end positions and well as the other settings for the camera is done with the LAS Core software (http://projects.biodiversity.be/ants).

For the comparison of the different techniques it was necessary to do the stacking with the same software package.

## Results

### Comparison of the focus stacking packages

To compare the times to calculate the extended focus image, there are two processes which need to be taken into account. First is the alignment of the images. This is an important process as images can be shifted in relation to one another, by movement of the camera or due to vibrations of the environment. During this alignment there is also a correction for the parallax effect. The second process is the actual combination of the several in-focus areas of the images into one in-focus image.

The pictures for both specimens were taken at a step-size of 40 µm, resulting in 74 and 41 pictures for *Meranoplus* sp. and *Trachys* sp. respectively. The resolution of the images on ‘Large’ is 5184 × 3456 pix. We used an aperture of f/4.5 and f/5.6 for the *Meranoplus* sp. and *Trachys* sp. respectively. For both specimens, the shutter speed was 1/100 s and ISO-100. The magnification of the 65 mm MP-E lens was 5×.

When looking at the results from the Google and Google Scholar search it is apparent that more people are mentioning Helicon Focus and CombineZP than the other two programs (Table 2). However, when performing a search on Flickr it becomes clear that the hobby and professional photographers prefer Zerene Stacker over the other software packages. Out of the four software packages tested, Auto-Montage is the least mentioned on the web and has no groups on Flickr. This is probably due to the difficulty of finding a trial version of the software. Moreover, the software is actually meant to be used by professional microscopy scientists.

### Auto-Montage

**Figure 2.**
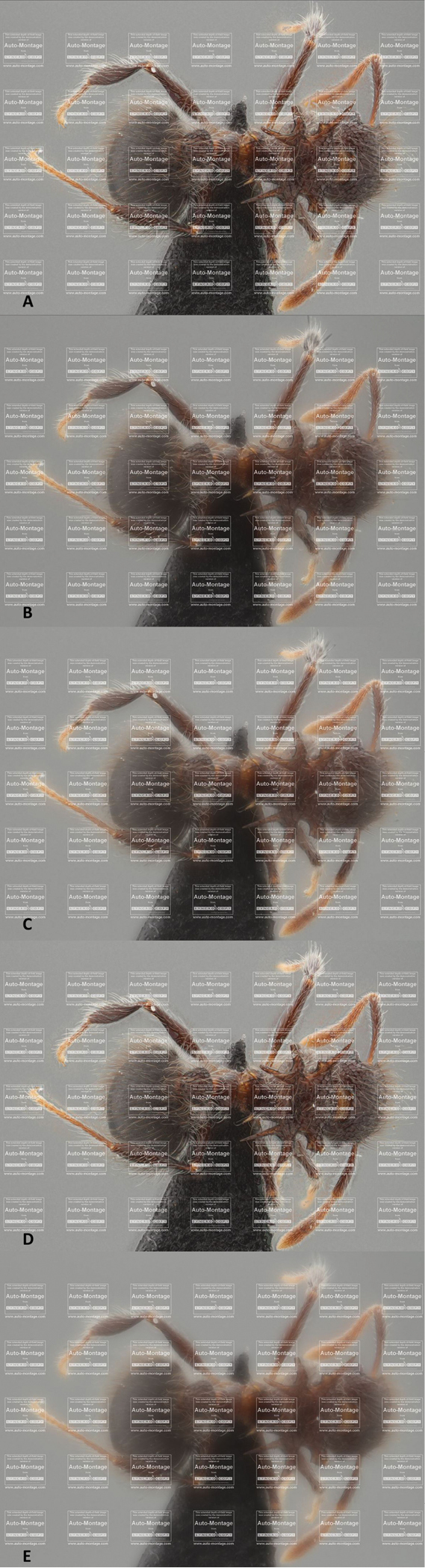
The results of the different stacking methods in Auto-Montage: each stacking option is provided with a single picture which is reduced in size by the Auto-Montage software itself and imprinted with watermarks. **A** represents the Blended Stacking option **B** picture composed by the Compound Weighted method **C** picture stacked by the Exponentially Weighted option **D** picture composed by the Fixed method and **E** picture stacked by the Weighted method.

Alignment of the images took 1 min 11 s and 41 s for the ant and the beetle respectively. For the *Meranoplus* sp., stacking functions 'Fixed' and 'Blended' are the fastest, by accomplishing the job in just under one minute, while the other stacking methods take more than twice that time (Table 1). For the *Trachys* beetle the same is found for the first and the last two stacking options. However the third option, the 'Weighted' method, is equally fast as the first two, delivering a result after approximately 35 s to 40 s. Although the alignment of the stack and stacking of these images is quite fast, none of the above available stacking methods provides a satisfying result. The main problems are the production of a substantial number of halos and the creation of a lighter area around the edges of the specimen. The most disturbing problem is found in the several weighted methods, in which the final picture appears as if it were shot through a translucent window (Figure 2).

**Table 1.** Comparison of the stacking time in the different software packages in default settings. * Auto-Montage is only able to stack 67 pictures on a workstation with the following parameters: Quad core 3.10 Intel i5 2400, 16 GB of RAM memory.

		*Meranoplus* sp.	*Trachys* sp.	Price
Auto-Montage*	Alignment	1'11"	0'41"	€ 2,500–3,000
	Fixed	0'56"	0'40"	
	Blended	0'58"	0'34"	
	Weighted	2'02"	0'37"	
	Exponentially Weighted	2'06"	1'27"	
	Compound Weighted	2'03"	1'25"	
CombineZP	Quick Alignment	1'40"	0'35"	Free
	Thorough Alignment	\	\	
	Do Stack	2'00"	1'13"	
	Do Soft Stack	2'03"	1'22"	
	Do Weighted Stack	5'30"	3'11"	
	Pyramid Weighted	2'49"	1'35"	
	Pyramid Do Stack	2'30"	1'31"	
	Pyramoid Maximum Contrast	1'42"	1'00"	
Helicon Focus	Method A (Average)	1'45"	0'47"	€ 108–225 (Premium)
	Method B (Depth Map)	2'10"	1'09"	
	Method C (Pyramid)	1'43"	0'57"	
Zerene Stacker	DMap (incl. alignment)	4'07"	3'15"	€ 89–283 (Pro: 3+: € 250 each)
	PMax (incl. alignment)	4'40"	3'00"	

### CombineZP

CombineZP has two options to align a stack, ‘quick alignment’ and ‘thorough alignment’. Unfortunately, we were only able to test the ‘quick alignment’ as the other option crashed the program every time. The alignment procedure took 1 min 40 s and 35 s for the *Meranoplus* and the *Trachys* specimen respectively (Table 1). Of the stacking procedures only the ‘Do weighted stack’ option is considerably longer than the other possible options, which are all well under three minutes for the *Meranoplus* sp. and max out at 1 min 35 s for the *Trachys* sp.

**Figure 3.**
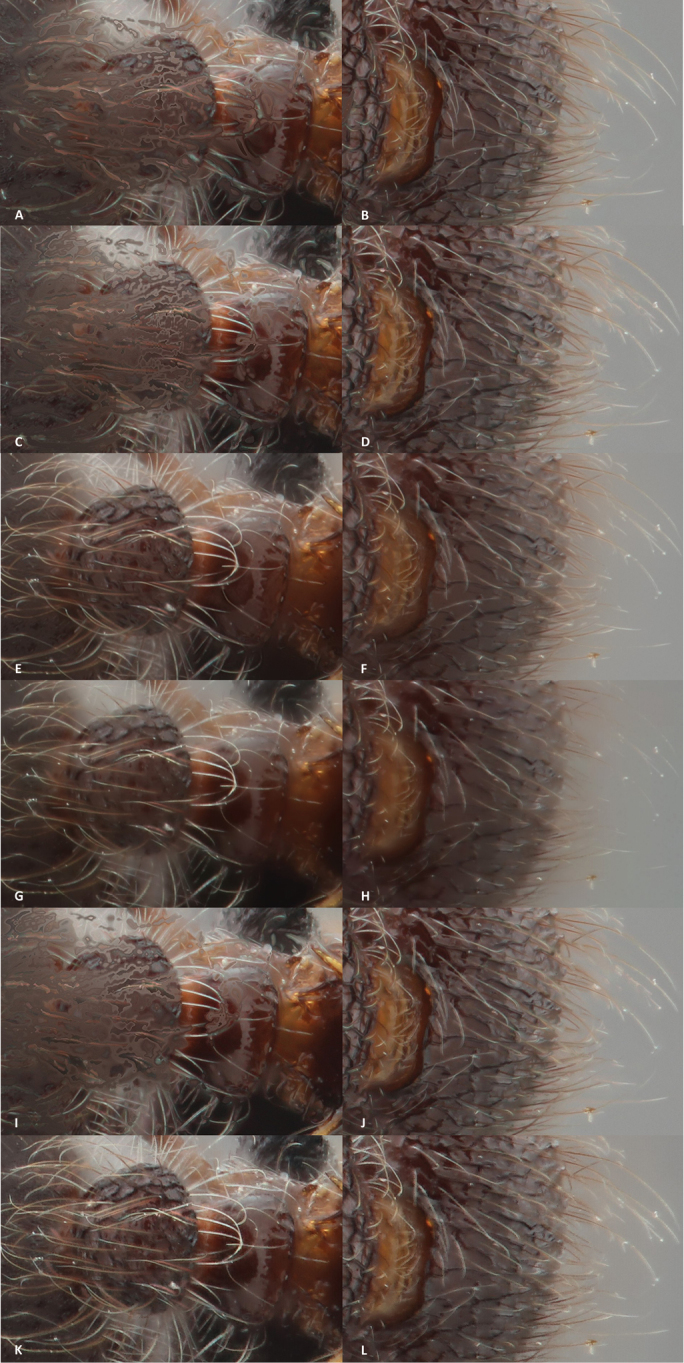
The results of the different stacking methods in CombineZP: each stacking option is provided with a detail of a hairy leg and a part of the head with hairs. **A, B** details of the stack pictures by the Do Stack method **C, D** details of the stacked picture with the Do Soft Stack **E, F** details of the Do Weighted Stack method **G, H** details of the combined pictured with the Pyramid Weighted method **I, J** details of the stacked image with the Pyramid Do stack method **K, L** Details of the Stacked image with the Pyramoid Maximum Contrast method.

At first sight the results look satisfactory. But when the different images are viewed at actual size, the results of the stacking itself is somewhat disappointing. The ‘Do stack’ (Figures 3A, B) and ‘Do Soft Stack’ (Figures 3C, D) only creates a few artefacts around the hairs on the head, although some halos do still occur around the hairs on the abdomen and on the thorax of the *Meranoplus* specimen. The pyramid weighted stack (Figures 3G, H) gives somewhat similar results, but the halos are more apparent. The only two methods that do not create halos are the ‘Do weighted stack’ (Figures 3E, F) and the ‘Pyramoid Maximum Contrast’ (Figures 3K, L). However, of these last two methods, the first creates a terrible light edge around the legs and other parts of the body, while the latter delivers a pixelated image when viewed at 100%.

### Helicon Focus

In Helicon Focus there are three options one can chose from to combine a stack of images. They all stack and align the pictures of the *Meranoplus* specimen in approximately 2 minutes, whilst the smaller stack of the *Trachys* specimen is produced in approximately one minute (Table 1). In the two first methods (the 'Average and Depth' methods) there is the possibility to change the parameters, whilst this is not possible for the 'Pyramid' method.

**Figure 4.**
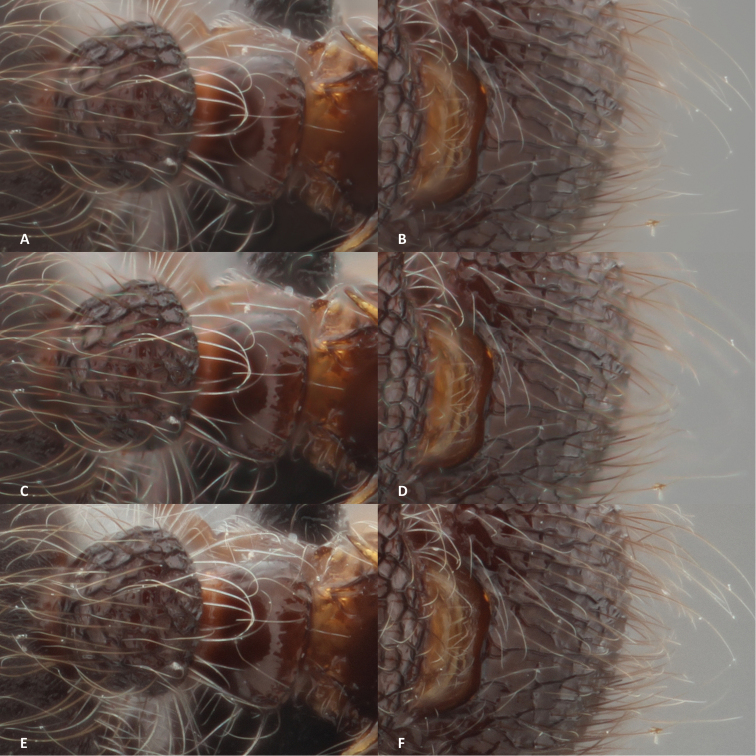
The results of the different stacking methods in Helicon Focus: each stacking option is provided with a detail of a hairy leg and a part of the head with hairs. **A, B** result of Method A (average) **C, D** result of Method B (depth) **E, F** result of Method C (pyramid).

Of the three methods available, the 'Pyramid' method (Method C, Figures 4E, F), as suggested by the guidelines of Helicon Focus, proved the most satisfactory. There are almost no halos present on the image and it has a clean look, but the brightness and the contrast is changed by the software. The 'Average' method (Method A, Figures 4A, B) produces a composed picture with a few halos around the abdominal hairs, but this method creates a lighter edge around the entire specimen. On another background this may work, but here it further distorts the image rather than accentuating it. The 'Depth' method (Method B, Figures 4C, D) is not suited for these types of specimens as the hairs produce halos all over the specimen. However, this can be controlled by adjusting the radius when choosing the depth method. We didn’t manage to find a set of parameters that gave a better result on this particular specimen. However, a specimen with less fine details, such as the beetle, would be fine with this method.

### Zerene Stacker

In Zerene stacker there are only two options to choose from, 'PMax', which is a pyramid stacking and 'DMap', a depth method. Both methods stack and align in a similar amount of time. The *Meranoplus* specimen is aligned and stacked in approximately 4 to 5 minutes, whilst the smaller stack of the *Trachys* specimen takes approximately 3 minutes to complete. In this software package it is also possible to change the parameters of the depth method.

**Figure 5.**
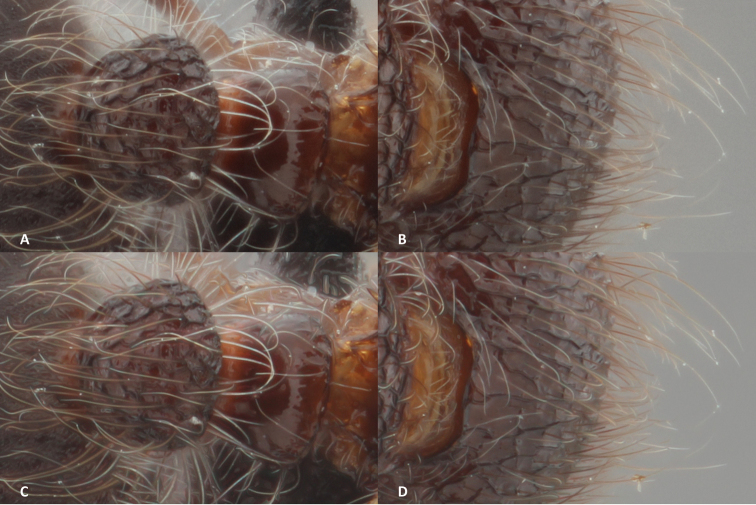
The results of the different stacking methods in Zerene Stacker, each with a detail of a hairy leg and a part of the head with hairs. **A, B** details of the stack pictures by the PMax method **C, D** details of the stacked picture with the DMap method.

Comparing the results (Figure 5) of the composed pictures, it is clear that the 'PMax' method works best. The image is well-composed, there are no halos, and details are clearly visible. Although only visible when viewing at full size, 'DMap' creates halos around the hairs on the back of the abdomen and around those on the head, and are most apparent around the hairs with another body part behind it, like a leg for example.

Taking into account all tests, we chose to process the pictures made by the stacking solutions with Zerene Stacker.

## Comparison of the high-end focus stacking solutions

### Leica MZ 16 A with DFC500

The pictures are the result of 70 images taken of the *Meranoplus* sp. and 41 images of the *Trachys* sp. respectively. These images are the result of a step-size of 36 µm and 39 µm respectively. Considering they are both approximately the same size, the step-size can be considered to be the same. Although the camera was able to take pictures with a resolution of 4080 × 3072 pix, the workstation connected to the set-up was not able to work at this resolution. The first resolution which successfully worked without affecting the quality of the pictures was 2040 × 1536 pix (Table 3).

**Table 2.** Comparison of the number of finds of analysed software: results obtained via Google and Google Scholar search engines and in the group search of Flickr.

	Google	Google Scholar	Flickr Groups
Auto-Montage	4K	2K	0
CombineZ	946K	1.7K	9
Helicon Focus	235K	1K	16
Zerene Stacker	114K	131	45

**Table 3.** Comparison of the settings.

	Resolution of images	Time to position specimen	Time for setting of stage	Time to set light conditions	Time to take pictures	Relative Price of the System
Leica MZ16A + DFC500	4080 × 3072	1–3 min	< 1 min	+/-5 min	1 image per 15 s	10–11
Leica Z6APO + DFC290	2048 × 1536	1–3 min	< 1 min	< 1 min	1 image per s	7–8
Canon-Cognisys	5184 × 3456	1–3 min	< 1 min	< 1 min	1 image per 2 s	1

**Figure 6.**
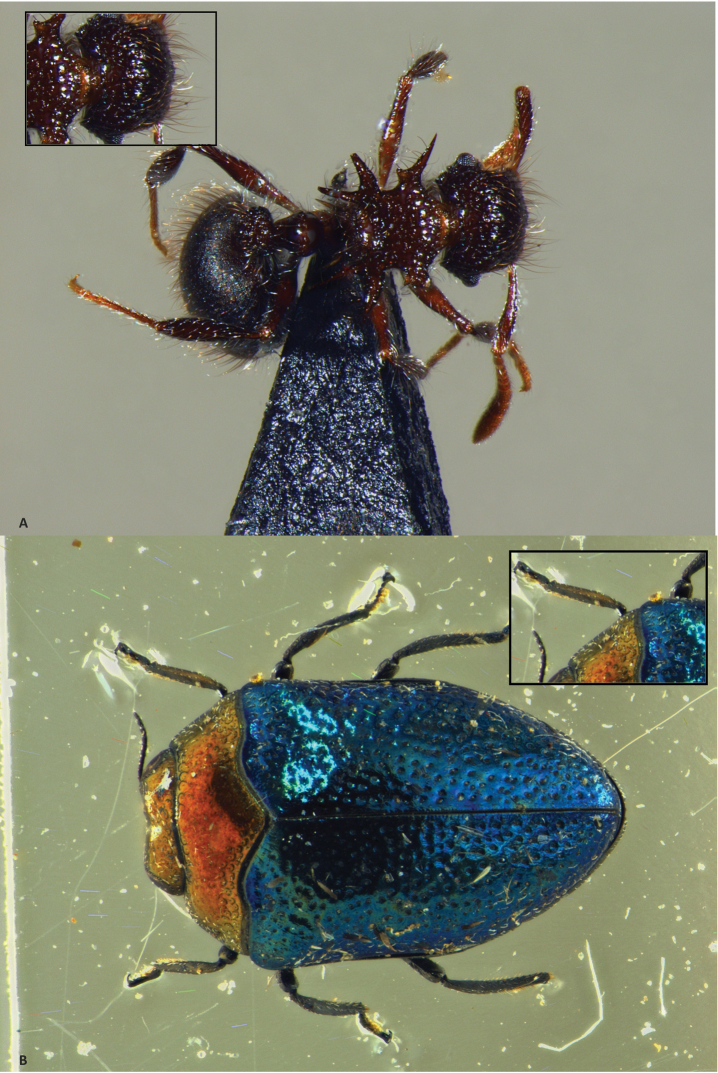
Focus stacking in Zerene Stacker. The small image in the upper corner provides a detailed close-up of 518 × 345 pix of the image at 100%. **A** Stack of 70 pictures, aligned and combined with PMax **B** Stack of 41 pictures, aligned and combined with PMax. The individual pictures of both stacks are made with the Leica MZ16A with DFC500 camera and Leica KL 1500 LCD lights.

The composed picture of the *Meranoplus* sp. could have benefited from a larger magnification (Figure 6). In this way more detail of the ant would be visible. However, judging the overall look of the picture, it is clear that there are parts which are over- and underexposed. The tips of hairs all appear shiny and reflect a lot of light. The overall coloration of the ant is dark brown-red.

The focus stacked image of the *Trachys* specimen is bright and clear, although it took a little bit more time to position the lights and the light diffusers. There is a small dark part on the elytra which is the result of the reflection of the black hole in the lens. This effect can only be solved with a sophisticated lighting setup involving axial lighting with beamsplitters. Without such a setup, this effect is unavoidable (Littlefield, pers. comm.). As the beetle doesn’t have a lot of fine details the picture looks better than the one of the ant. But again fine details are not visible because of the small picture dimensions. Purchasing this set-up will cost you approximately 10 Canon-Cognisys systems.

### Leica Z6 APO with DFC290

As we set more or less the same step-size for the *Meranoplus* sp. and the *Trachys* sp. while making the separate images (39.68 µm and 39.19 µm respectively) this resulted in 77 and 44 images for these two specimens. The camera provides HD pictures with a resolution of 2048 × 1536 pix (Table 3).

**Figure 7.**
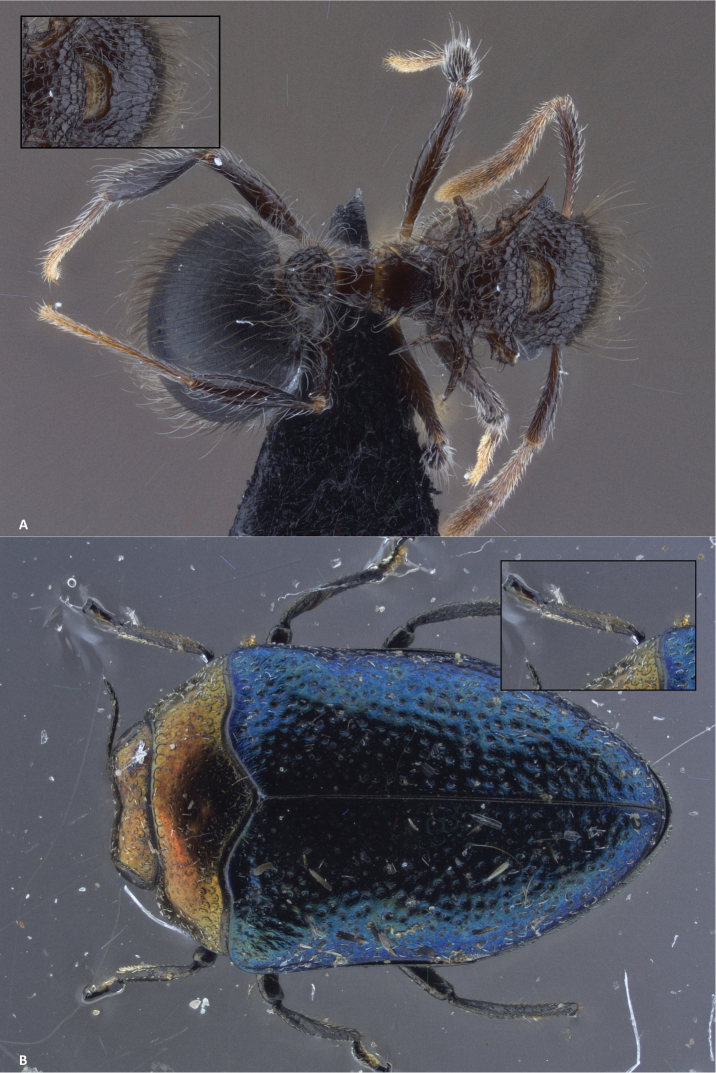
Focus stacking in Zerene Stacker. The small image in the upper corner provides a detailed close-up of 518 × 345 pix of the image at 100%. **A** Stack of 77 pictures, aligned and combined with PMax **B** Stack of 44 pictures, aligned and combined with PMax The individual pictures of both stacks are made with the Leica Z6 APO with DFC290 and Manfrotto led light system.

The resulting picture (Figure 7) shows a well-illuminated and detailed specimen. There are no areas which are over- or underexposed. The ant itself has an overall dark blackish coloration with light highlighted hairs. The only downside is that the resolution doesn’t offer more detail than is visible on a regular (21 inch) computer screen.

The *Trachys* specimen is quite dark on the elytra making it difficult to see details in that area. Aside from the illumination issue, the picture is sharp and there are no parts that are overexposed. Again it is unfortunate that the image dimensions aren’t larger, because this would aid in viewing details on legs and antennae. The set-up we tested here can be purchased for approximately eight Canon-Cognisys set-ups.

### Canon-Cognisys set-up

**Figure 8.**
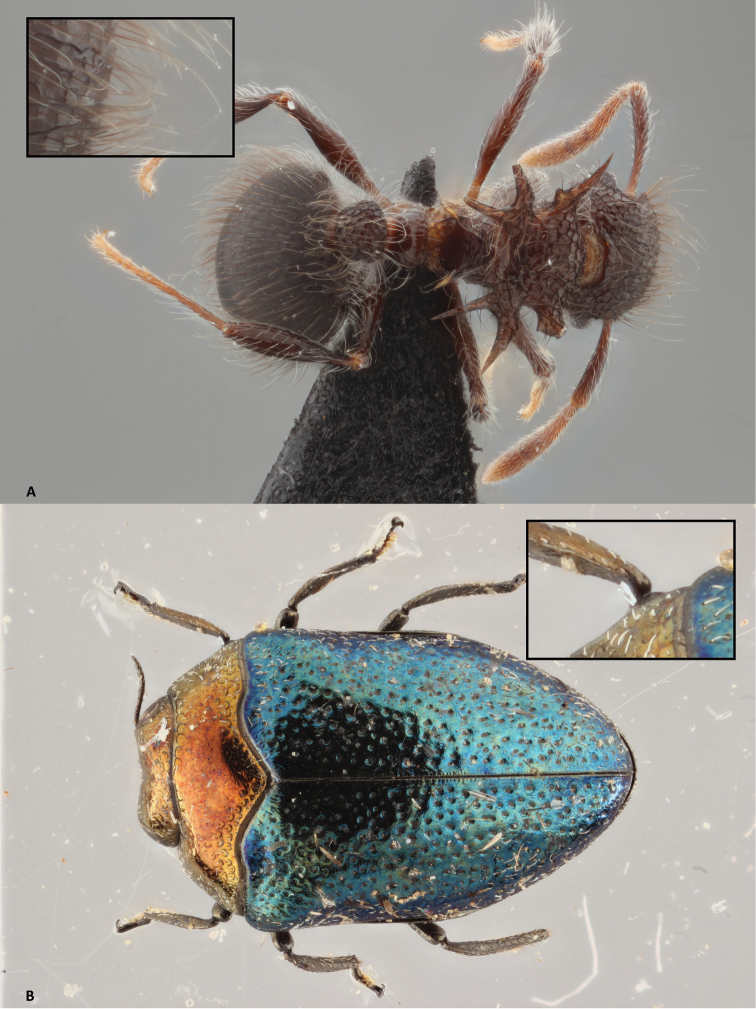
Focus stacking in Zerene Stacker. The small image in the upper corner provides a detailed close-up of 518 × 345 pix of the image at 100%. **A** Stack of 74 pictures, aligned and combined with PMax **B** Stack of 41 pictures, aligned and combined with PMax. The individual pictures of both stacks are made with the Canon-Cognisys set-up and double flash lights.

Using the double flash lights inside a light chamber creates a smooth light resulting in even illumination without any over- or underexposed parts as can be seen in both the *Meranoplus* and the *Trachys* specimens (Figure 8). Apart from the easy illumination, the large benefit is the large image dimensions provided by the Canon 600D. Fine details such as the hairs on the ants’ legs, head and abdomen can be clearly seen. The set-up we presented here costs approximately € 3,000 (Table 3).

## Discussion

### Comparison of the software packages

All the different software packages tested compose (aligning and stacking) a new picture in three to four minutes, except for Helicon Focus, which does the job in half the time. Lowering the values of the alignment parameters or even unchecking this option in Zerene Stacker, will reduce the stacking time as well. To speed up the stacking process there are many parameters available in the professional version of Zerene Stacker, which can lower the process time by a factor of three when altered (Littlefield 2014). However, in CombineZP, there are some settings which make the process last slightly longer as with the 'Do Weighted Stack' method. The two stacking methods of Zerene Stacker and those of CombineZP are slower than the ones from Helicon Focus and Auto-Montage. But time isn’t the most important factor as the computing can be done after working hours. In the end the quality of the focus stacked picture is what really matters: in these tests both Helicon Focus and Zerene Stacker provided the best results and both have more or less the same price. Helicon Focus has the possibility to make a 3D model made out of the information available in the image stack. This might be an interesting feature, however it has little scientific value as it only delivers a decent model with objects that are smooth and does not demonstrate fine structures such as insect legs. In fact as Zerene Stacker is also able to compute depth maps, but it is also possible to make similar models using third party software. Besides depth maps, Zerene Stacker can make stereo and ‘rocking’ pictures (gifs) which give the impression of 3D when viewed cross-eyed for the stereo option. A huge benefit of both Helicon Focus and Zerene Stacker is that they can control a StackShot through their own interface. This might be useful when the stacking is done immediately after taking the pictures. However, many more images will be stacked during a working day when processed as a batch file. Helicon Remote, bundled within the premium edition of Helicon Focus, also enables the direct control of an auto-focus lens, when the attached DSLR is a fairly recent one (for the exact list see the Helicon Focus web page). Other third-party software also exists for Zerene Stacker or other focus stacking software (ControlmyNikon (www.Controlmynikon.com); Magic Lantern (www.magiclantern.fm)). Both of these packages have easy tools to retouch the final image when necessary, but Zerene Stacker’s tools are more extensive. We did not use this option as we wanted to see the results before the actual retouching; better raw results need less time retouching afterwards. The only disadvantage of the software packages, might be that Helicon Focus is only able to deal with a stack of less than 255 pictures. But as mentioned before, unless the object is quite straightforward, without any fine details, it is better to make substacks when dealing with such a large amount of pictures. This will take more time to process a stack, although it is also possible to do this during the night as a batch file. Overall the two software packages deliver the same results, although we have seen that Helicon Focus sometimes creates a few more halos than Zerene Stacker in certain cases. Given the more or less similar end result one might benefit from purchasing both software packages as Zerene Stacker has more retouching abilities and Helicon Focus can stack images faster. This is more or less visible in the internet search results as well; researchers are going for the fast processing of Helicon Focus, while professional/hobby photographers chose Zerene Stacker because of the ability to manipulate each step during and even after the focus stacking.

### Comparing the high-end focus stacking solutions

There is a large difference in sensor size between the three systems (Table 4). The sensor size of the Canon CMOS sensor on the D600 makes it possible to fill the sensor with an object of 22 mm to 4.4 mm size depending on the magnification of the MP-E lens (1× to 5× respectively). The two Leica systems are able to fill the sensors of their cameras with an object measuring 0.9 mm and 0.6 mm for the MZ16A and Z6APO, respectively.

**Table 4.** Sensor size versus magnification of the systems tested.

	Leica MZ16A + DFC500	Leica Z6APO + DFC290	Canon 600D + MP-E 65 mm
Magnification	16:01	06:01	05:01
Objective used	0.63	2	\
Resulting magnification	10	12	5
Sensor width (mm)	8.8	6.6	22.3
Pixels (width)	4080	2048	5184
# pixels/mm	4674	3318	1162
# pixels/µm	4.67	3.32	1.16
# µm/pixel	0.21	0.3	0.86
Sensor filling (mm)	0.87	0.62	4.46

However, when an object is pictured that is sensor-filling on the Canon CMOS sensor, the Canon-Cognisys set-up is able to deliver a picture more than twice the size of the other two techniques. The Leica MZ16A would perform better with a adequate processing power and memory. In that case the difference in resolution wouldn’t be as apparent, but would still be noticeable (5184 × 3456 to 4080 × 3072). When the final stacked picture is sharp, even with less resolution, it will still be suitable for a publication. However, it will not be possible to see more detail by enlarging the picture although this is possible with higher resolution pictures.

The time needed to make the set of pictures on the Leica MZ16A was substantially longer than with the other two techniques. This might of course be due to the difference in computing power and perhaps the older camera in this set-up.

Another difference between the different approaches is the lack of a good lighting set-up with the MZ16A. Using two microscope lights is far from perfect to obtain a smooth illumination. However this can be solved by using a similar light as used in the Z6APO set-up or perhaps even a standard Leica solution as the 5000 LED dome. But again as this set-up is already the most expensive of the three tested, one might chose a more budget-friendly approach or go for another high-end solution. The light used with the Z6APO and the Canon-Cognisys deliver more or less the same results, although they are two complete different approaches as one is continuous lightning and the other is flash light. Looking at the colour of the specimens on the different images, there appears to be a problem: none of them actually has the same colour composition, while all of them were calibrated with a grey card.

**Figure 9.**
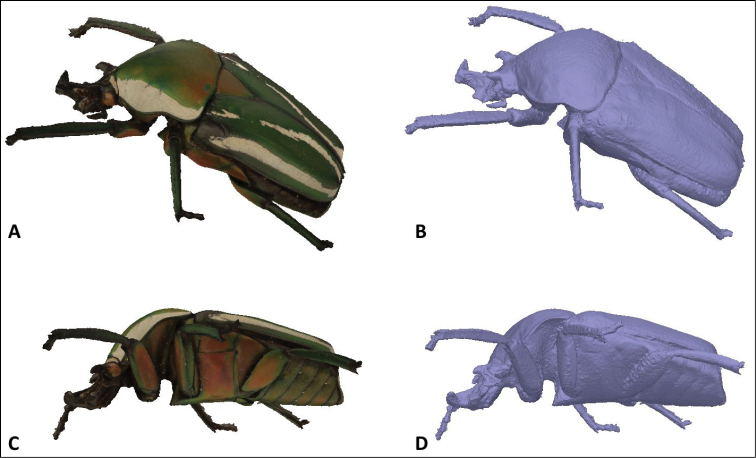
A 3D model of a *Dicranorrhina* sp. beetle. The left pictures (**A, C**) represent the 3D model with its texture, while the right pictures (**B, D**) are from the model with the mesh only and show the level of detail of the 3D model made in Agisoft Photoscan.

One might argue why should you bother with taking focus stacked pictures as photogrammetry enables the creation of 3D models of insects (Nguyen et al. 2014). We tried this approach as well, although we used a different software package, Agisoft Photoscan (www.agisoft.com). Previously the software had had difficulties in aligning images with a low depth of field. Recent software updates made the software package stronger and making insect 3D models is no longer an issue (Figure 9). However, looking at the details provided by these models, both with and without texture, they are far from detailed, even with the better resolution provided by the Agisoft Photoscan software compared to the BOB Capture models in Nguyen et al. 2014 (Model of a longhorn beetle similarly-sized to our *Dicranorrhina* beetle: http://dx.doi.org/10.4225/08/531E573D7F06C) (3DSOM is now incorporated in BOB Capture, www.bigobjectbase.com/bob-capture). Areas with hairs or transparent parts lack any detail. In fact the only way to get a decent 3D model of an insect is by µCT scanning. Photogrammetry works very well in other fields of research (Mathys et al. 2013a, 2013b) but for species recognition and determination it is not precise enough, although they could be great educational models to show on websites or in museum exhibitions. Therefore we think that focus stacking is still an appropriate way to digitize entomological specimens, as it delivers detail which scientists need for their research.

## General conclusion

Based on the results presented we can conclude that the Canon-Cognisys set-up as we currently use it delivers results that are equal, or even better, than high-end approaches. This merely is due to the simple lighting set-up, the high resolution, and the low noise delivered by the Canon DSLR. All this combined makes it possible even for untrained people to take good quality pictures. The fact that everything is easy to replace when better cameras or lenses become available is a huge advantage of this set-up. By changing lenses (60 mm Macro or 100 mm Macro) it is also possible to photograph specimens of 10 cm to 20 cm (e.g. butterflies, spiders, scorpions, even minerals), which show large depths and benefit from focus stacking. Preliminary tests show that even specimens in liquids (alcohol, glycerine, etc.) can be photographed without the need to change the set-up. Moreover, the low cost for the entire set-up enables the use of it for mass digitization as multiple packages can be purchased and operated simultaneously, which will considerably speed up the amount of specimens digitized. After a few months of testing in a mass digitization context, we are able to easily generate focus stacked images of 50 specimens a day, when only one view is needed or 16 specimens when three views per specimen is necessary (picture of the labels and processing of focus stacked picture included). Within a full-time contract one person can process 10 000 specimens in 50 weeks with a cost of approximately € 4.30 per specimen or per view (picture of the labels included). We expect that the cost per specimen will decrease and the amount of specimens photographed a day will increase once the workflow becomes more fluid.

The huge challenge for the future will be managing the load of data produced by the stacking process, keeping track of all the metadata created, and providing it in an automated way online.

When needing to decide upon the software packages one might be attracted to the freely available software package CombineZP. The results are satisfactory when the picture itself has a low number of pixels (for instance those produced by the Leica DFC290) and the use is for a printed publication where the resulting picture is smaller than its actual size. In this case the software could be a temporary solution. However, as these days full-size high pixel images are easy to download, one has to choose the best results especially when Helicon Focus and Zerene Stacker deliver considerably better results and only cost approximately € 250 for the full packages with permanent licenses.
